# The unveiled face of IEI: Children Cancer Hospital—Egypt (CCHE-57357) experience

**DOI:** 10.3389/fimmu.2025.1570328

**Published:** 2025-10-30

**Authors:** Nesrine Radwan, Youssef Medany, Hanaa Rashad, Ahmed Elhemaly, Mahmoud Hammad, Hany Abdel Rahman, Mona Fakhry, Nora Mahmmoud Marouf, Ahmed Mahdy, Mariam Elsherif, Maram Farouk Salama, Nesreen Ali, Sally Talaat, Seham Gohar, Ahmed Emad, Iman Sidhom, Nahla EL-Sharkawy, Alaa El-Haddad

**Affiliations:** ^1^ Pediatric Allergy, Immunology & Rheumatology unit, Children’s hospital, Ain Shams University, Cairo, Egypt; ^2^ Pediatric department, Children Cancer Hospital Egypt (CCHE-57357), Cairo, Egypt; ^3^ Pediatric Oncology Department, National Cancer Institute, Cairo University, Cairo, Egypt; ^4^ Clinical Pathology Department, National Cancer Institute, Cairo University, Cairo, Egypt

**Keywords:** inborn error of immunity, malignancy, leukemia, lymphoma, immunodysregulation, bone marrow transplantation

## Abstract

**Background:**

Inborn errors of immunity (IEI) are a heterogeneous group of different disorders characterized by a defect in the function and/or components of the immune system. Malignancy is the second common cause of death following recurrent infections.

**Aim:**

We present our experience in Children Cancer Hospital Egypt (CCHE-57357) in diagnosing IEI patients who first presented with malignancy rather than infections.

**Methods:**

Data of 19 IEI patients with malignancy referred to the immunology clinic was collected. The reasons for referral were stunted growth or presence of bronchiectasis at presentation, persistent eczema, significant chemotoxicity, history of recurrent infection either during or after stoppage of chemotherapy, and relapse of lymphoid malignancy after auto-BMT.

**Results:**

The patients comprised 14/5 men/women. Their median age at diagnosis with malignancy was 7 years (1.5–16 years). In addition, 13/19 had lymphoma (Hodgkin’s/non-Hodgkin’s) and 6/19 patients had leukemia. Moreover, 9/19 had history of repeated infections, 4/19 had failure to thrive, 5/19 had clubbing, 4/19 had bronchiectasis, 3/19 had significant chemotoxicity, 8/19 had low immunoglobulin, 12/19 had abnormal lymphocyte subsets, and 3/19 had a relapse of the original disease. Genetic testing was done to 18/19. The diagnoses based on genetic and/or immunological investigation according to the IUIS classification were 7/19 (37%) immune-dysregulation, 4/19 (21%) combined immunodeficiency with syndromic features, 3/19 (15.7%), combined immunodeficiency, 3/19 (15.7%) predominantly antibody defect, and 2/19 (10.5%) bone marrow failure defect.

**Conclusion:**

Collaborative work between immunologist and oncologist helped in diagnosing patients with IEI who first presented with malignancy.

## 1 Introduction

Inborn errors of immunity (IEIs) are a group of inherited diseases that are caused by damaging germline variants in single genes affecting the immune cell function and/or number. IEIs have a wide range of clinical picture. Patients usually present with an increased susceptibility to infections (1), and they can also present with autoimmune manifestation, autoinflammation, allergy, bone marrow failure, and/or malignancy (1). This broad clinical picture causes the patients to present to a wide array of providers ranging from primary care physicians to various pediatric subspecialists (2).

Malignancy in IEI patients is a leading cause of mortality following infections. There is a relatively higher risk of mortality due to either disease-related or treatment-related causes in comparison to other patients, and this risk varies between different malignancy subtypes (3–5). It is reported that malignancy can develop in 1.5%–25% of IEI patients, which is considered a relatively high incidence (6, 7). Any type of malignancy can occur, but the most common is lymphoma, representing around 60% of cases, with non-Hodgkin’s lymphoma (NHL) being the greatest contributor (8–10).

This high incidence of malignancy is attributed to both intrinsic and extrinsic factors that initiate and progress the malignant transformation (11–14). Intrinsic factors include various factors such as errors in cell apoptosis, accelerated immune senescence, abnormalities involving cell development and/or signaling, actin cytoskeleton, cytotoxicity, DNA repair, chromosome instability, and telomere maintenance (11, 12, 14, 15). Extrinsic factors include chronic tissue inflammation and numerous infectious agents associated with oncogenic viruses such as Epstein–Barr virus (EBV) in lymphoproliferative conditions and soft tissue tumors, human papillomavirus (HPV) in epithelial tumors, and *Helicobacter pylori* in stomach cancer (13, 16).

Recurrent infections are usually the main presentation of IEIs. However, some patients may present with malignancy as a first clinically noted presentation, especially in patients with delayed onset or mild IEI phenotypes. The early onset of cancer or a recurrence of lymphoma should raise the suspicion of an underlying IEI (17–20). In a French national registry that retrospectively followed 1375 IEI patients for 10 years. At the beginning of the study, 37% of patients suffered from noninfectious complications, and it was increased by an additional 20%. Malignancy occurred in 7% of patients. A total of 14% of the patients died during the study; 43% of those deaths were due to noninfectious events, of which one quarter was due to malignancy (21).

Treatment of malignancy in IEI patients is challenging as they respond differently to cancer treatment. They can experience increased toxicity and/or decreased efficacy of standard cancer treatment, and unfortunately it is very difficult to predict such events, even within a single IEI subtype. They also suffer from an increased risk of malignancy recurrence (3). Albeit it was found that some children with co-existent IEIs and malignancy might benefit from adjustment in the treatment plan such as dose modifications of chemotherapy, avoidance of radiotherapy, use of antimicrobial prophylaxis, or immunoglobulin replacement therapy (5). But there are no clear guidelines for that. Conversely, cancer recurrence poses the greatest threat in some IEI patients, and accordingly it may not be desirable for dose reductions to be uniformly applied. Hematopoietic stem cell transplantation (HSCT) should be considered in some patients based on diagnosis and medical condition. The aim of HSCT is to achieve both malignant disease control and treatment of the underlying IEI. However, before any of these treatment modifications can be implemented, the diagnosis of IEI must have been considered and confirmed by clinical, laboratory, and genetic testing (3, 21).

Data are rare about patients who first present with malignancy, and the presence of a diagnostic workflow for those patients is still not well developed. Bosch JVWT et al. (3) suggested a diagnostic workflow to be used to help in the diagnosis of those patients. It included a careful review of the patient’s history and physical examination, combined with careful use of routine laboratory investigations and pathological review aiming at identifying patients who warrant additional investigation.

In this study, we aimed to present our experience in Children Cancer Hospital Egypt (CCHE-57357) in diagnosing IEI patients who first presented and were diagnosed with malignancy rather than recurrent infections.

## 2 Methodology

This is a retrospective descriptive study conducted on patients having malignancy and referred to the immunology clinic in Children Cancer Hospital Egypt (CCHE-57357) for suspicion of an underlying IEI. The causes of referral were either one or more of the following: history of repeated severe infections during or after chemotherapy, presence of bronchiectasis, clubbing or stunted growth at presentation with malignancy, and recurrence of lymphoid malignancy after auto-bone marrow transplantation. The inclusion criteria refer to patients who presented with malignancy and were found to have an underlying IEI by genetic sequencing and/or specific confirmatory laboratory tests. The exclusion criteria refer to patients known to be with IEI and who developed malignancy as patients with ataxia telangiectasia.

All patients were subjected to the following: (1) provide a detailed history, which included age at recruitment, age at diagnosis with malignancy, parental consanguinity, living town, family history of malignancy or recurrent infection specifically the siblings, and history of any of the 10 warning signs before and after chemotherapy; (2) clinical examination was done, and it included anthropometric measures and general examination with stress on the presence of clubbing, organomegaly, eczema, or any peculiar facies; and (3) laboratory examination was done at CCHE-57357, and it included complete blood count with differential and serum immunoglobulin levels (IgG, IgM, and IgA) by nephelometry and flow cytometry assessment for lymphocyte subsets that included both absolute and lymphocyte count. Immunological labs were done before starting chemotherapy and at least after 6 months of stopping the treatment. The lymphocyte subsets included CD3, CD4, CD8, CD19, CD56, CD45RO, CD45RA, CD19+ CD27+ Ig D- (class-switched memory B cells), and CD19+ Ig D+ CD27- (naïve B cells). The flow cytometry specimens include fresh EDTA and heparin blood used for lymphocyte subset enumeration. Mononuclear cell separation was done using Ficol separation technique. It includes analysis for CD3, CD4, CD8, CD16, CD19, CD20, CD45, CD56, CD45RA, CD45RO, CD27, and IgD; the fluorochromes used for staining are as per panel. The samples were analyzed by using multicolor flow cytometry (Coulter Navios EX). Then, Kaloza software was applied for analysis. Whole exome sequencing (WES) was done either in commercial labs or institutional labs.

A consent form was obtained from the children’s legal guardian, and ethical approval was obtained from the Institutional Review Board (IRB) at the Children’s Cancer Hospital—Egypt (CCHE/57357), IRB #N-0059–015-025.

## 3 Results

The immunology clinic was established in CCHE-57357 in November 2018. During the period 2018–2023, 283 patients were referred to the immunology clinic because of one of the following reasons: (a) known IEI patients who developed malignancy and under chemotherapy treatment for regular immunological follow-up, (b) patients with non-malignant lymphoproliferation for possibility of an underlying immunological disease, (c) patients diagnosed with malignancy who developed recurrent infections during chemotherapy or after stoppage of chemotherapy, (d) patients who had recurrence of lymphoid malignancy after auto-BMT, (e) those who had peculiar presentations such as clubbing, stunted growth, and bronchiectasis at the time of malignancy diagnosis for possibility of underlying IEIs, and (f) patients with a history of sib death due to either malignancy or recurrent infections.

**Figure 1:**
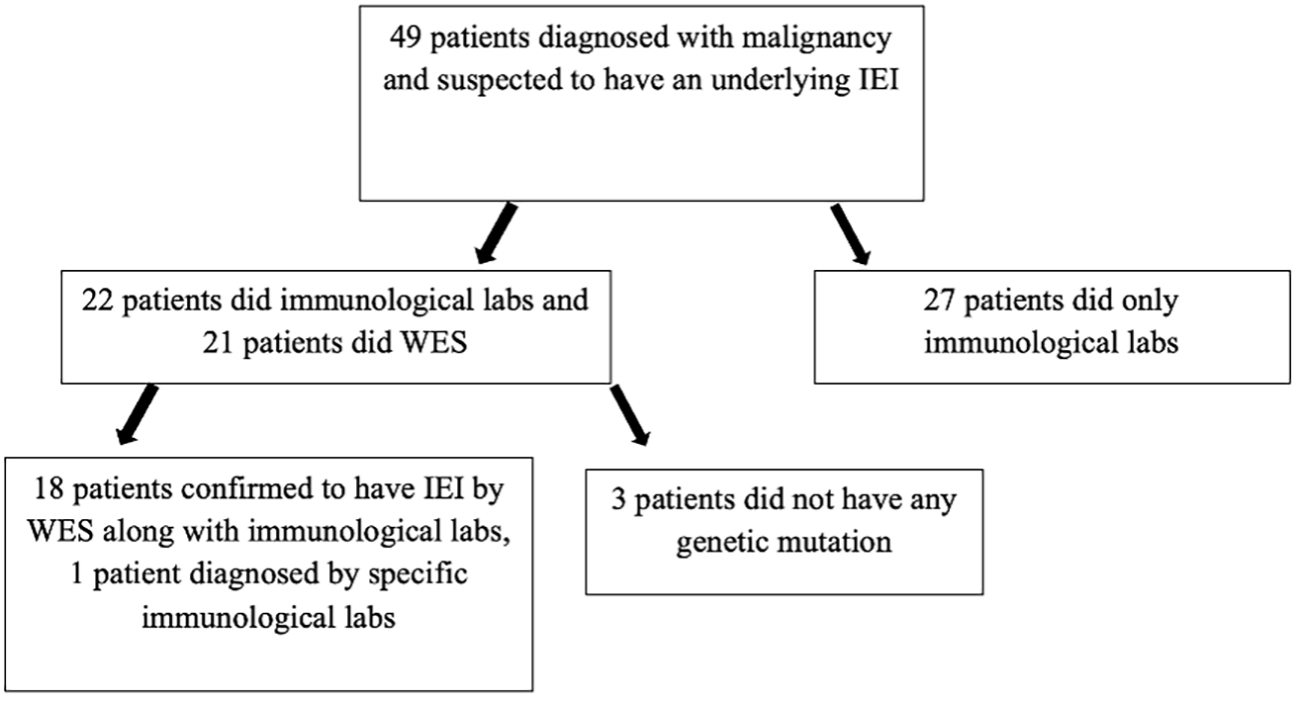
Patients diagnosed with malignancy and who were suspected to have an underlying IEI. WES, whole exome sequencing.

Out of the 283 patients, 49 patients were suspected to have an underlying IEI based on a history of recurrent infections during their follow-up or peculiar presentation at diagnosis of malignancy or positive family history. The confirmation of IEI diagnosis in 19 patients was based on mainly at least two of the following conditions: genetic sequencing along with clinical phenotype and immunological laboratory. Three patients were suspected to have IEI based on a history of recurrent severe infections during chemotherapy, low CD19, and low IgG, but genetic sequencing did not show any mutation. A total of 19 patients were confirmed to have an underlying IEI. The diagnosis of IEI was not confirmed in the remaining 27 patients as the clinical phenotype and basic immunological labs done were not enough to confirm a final diagnosis on whether it was a primary or a secondary immunodeficiency (**Figure 1**).

Those 27 patients had the following clinical phenotypes: (a) recurrent infections (recurrent abscesses, recurrent bronchopneumonia, and invasive fungal infections), (b) autoimmune manifestation following chemotherapy as vasculitis, immune thrombocytopenia, nephritis, and arthritis, and (c) peculiar clinical and radiological findings at presentation with malignancy (bronchiectasis, eczema, clubbing, and failure to thrive). The immunological labs showed the following: 10 patients had abnormal lymphocyte subsets, and eight patients had low immunoglobulin levels. A family history of recurrent infections in one of the family members was established in four patients, one patient had sib death early in life due to infection, and two patients had multiple family members diagnosed with malignancy.

Our cohort of 19 patients proven to have an underlying IEI after presenting with malignancy included 13 (69%) men and six (31%) women. Their median age at diagnosis with malignancy was 7 years (1.5–16 years), and the median age of referral to the immunology clinic was 8 years (4–19 years). A total of 11 patients had positive parents’ consanguinity. A history of repeated infections was present in 9/19, 4/19 had failure to thrive, 5/19 had clubbing, 4/19 had bronchiectasis, 3/19 had significant chemotoxicity, and 3/19 had a relapse of the original disease (detailed clinical and laboratory data are shown in **Table 1**).

**Table 1:** Detailed clinical and laboratory data of the patients.

Patient number	Gender	Age at presentation, in years	Age at diagnosis, in years	Type of malignancy	EBV positivity	Positive 10 warning signs	Other features	Immunoglobulin level	Lymphocyte subset	Genetic mutation	Positive family history of malignancy/immunodysregulation/sib death	Treatment adjustment	Outcome
Lymphoma patients													
P1	F	3	8	Hodgkin’s lymphoma	negative	–	Patient relapsed after first remission, significant chemotoxicity	Normal IgG, IgM, IgA	CD4 low	ITK gene mutation Chr 5:156635997: c.236C>T: p.Pro79Leu, Chr 5:156670644: c.1072A>G: p.Ile358Val (missense variant)	2 sibs died from Hodgkin’s lymphoma	IVIG	Dead
P2	M	7	9	Burkitt’s lymphoma	Positive	Recurrent pneumonia, recurrent sinus, prolonged need for antibiotic (after chemotherapy)	Bronchiectasis after chemotherapy	Low IgG, IgM, IgA	Low CD19	Splice region mutation in SH2D1A, c.201 + 3A>G	Negative	IVIG, refused HSCT	Alive
P3	M	8	9	Burkitt’s lymphoma	Positive	Recurrent pneumonia, recurrent sinus, prolonged need for antibiotic (after chemotherapy)	Bronchiectasis after chemotherapy	Low IgG, IgM, IgA	Low CD19	X-linked nonsense mutation in SH2D1A, c.163C>T, p.R55X	Negative	IVIG, did HSCT	Dead
P4	F	9	13	Hodgkin’s lymphoma	negative	Recurrent pneumonia (after chemotherapy)	Significant chemotoxicity Dry scaly skin, short stature, clubbing, bronchiectasis at presentation, conical teeth	Normal IgG, IgM, IgA	Low CD4, 19	ITK homozygous chr5:156667071 G>A; c.852-1G>A	Positive sib with same mutation who developed HL	IVIG, refused HSCT	Alive
P5	F	10	11	Hodgkin’s lymphoma	negative	–	–	Normal IgG, IgM, IgA	normal lymphocyte subset	ITK homozygous chr5:156667071 G>A; c.852-1G>A	Positive sib with same mutation and HL	refused HSCT	Alive
P6	M	15	16	Hodgkin’s lymphoma	positive	–	Eczema, warts over both wrists, persistent thrombocytopenia since birth, normal platelet volume, normal WAS protein expression	Low IgG, IgM, IgA	Low CD19	WASp mutation: c.763del, p.Gin255ArgfsTer6	Negative	Relapsed on chemotherapy	Alive
P7	M	2	5	Burkitt’s leukemia	N/A	Recurrent pneumonia, recurrent sinus, prolonged need for antibiotic, failure to thrive (before chemotherapy)	Significant chemotoxicity and recurrent severe infections during chemotherapy, clubbing	Normal IgG, IgM, IgA	Low CD19	KRAS missense mutation: p/ALA146 Thr,c.436G>A likely pathogenic heterozygous and PIK3CD p.Val3070Met, c.1108G>A) heterozygous VUS	Negative	IVIG, waiting for HSCT	Alive
P8	M	5	10	Hodgkin’s lymphoma	Positive	–	Recurrent minor chest infection (before chemotherapy), bronchiectasis at presentation, clubbing	Normal IgG, IgM, IgA	Low CD19	TNFRSF9:Homozygous c.325G>A, p.Gly109Ser	Recurrent sib death due to chest infection	IVIG, did HSCT	Alive
P9	M	1.5	4	Burkitt’s lymphoma	Positive	–	–	Low IgG, IgM, IgA	Low CD19	SH2D1A gene mutation (details N/A)	Recurrent sib death due to malignancy	IVIG, did HSCT	Alive
P10	M	16	19	Hodgkin’s lymphoma	Positive	–	–	N/A due to chemotherapy	N/A due to chemotherapy	RASGRP1 homozygousc.1238T>C;p.Leu413Pro	A sib with alopecia, anemia, and immunothrombocytopenia with same mutation	Did HSCT	Alive
P11	F	10	11	Hodgkin’s lymphoma	N/A	Recurrent chest infection (after chemotherapy)	–	Normal IgG, IgM, IgA	Low CD19	Heterozygous variant of unknown significance NFKB1 c.1822G>A, p.D608N	A sib who died from HLH and recurrent infection	Did HSCT	Alive
P12	F	5	8	Hodgkin’s lymphoma	Negative	–	Relapse after auto-BMT	normal IgG, IgM, IgA	Low CD19	heterozygous mutation TP53:c.818G>A;p.Arg273His)	Negative	Did HSCT	Alive
P13	M	6	16	Burkitt’s lymphoma	Negative	–	Relapse after auto-BMT	N/A due to chemotherapy	Low CD19	heterozygous mutation TP53:c.743G>A;p.Arg248Gln)	Negative	Pending HSCT	Alive
Leukemia													
P14	M	5	6	Acute myeloid leukemia	N/A	Recurrent chest infection, failure to gain weight (before chemotherapy)	Chronic diarrhea, clubbing	Low IgG, IgM, IgA	Low CD4,19	MSH6:c.453dupT; p.Thr152Tyrfs*20 (frameshift)	Recurrent sib death due to chest infection	IVIG	Dead
P15	M	8	13	Acute myeloid leukemia	N/A	–	Severe GVHD after allo-BMT, recurrence of lymphadenopathy	Normal IgG, IgM, IgA	Normal lymphocyte subset	FAS homozygous VUS C.908T>G (p.Leu303Arg)	A sib with same mutation	Sirolimus, refused allo-BMT	Alive
P16	F	5	8	Acute myeloid leukemia	N/A	Recurrent ear infection and abscesses, need for prolonged of antibiotic (after chemotherapy)	Eczema, coarse features	Normal IgG, IgM, IgA, elevated IgE	Normal lymphocyte subset	Pending genetics	Sib with elevated IgE and eczema, and coarse features	Anti-IL13,4	Alive
P17	M	9	12	Acute lymphocytic leukemia	N/A	Recurrent pneumonia, recurrent sinus, prolonged need for antibiotic, failure to thrive (before chemotherapy)	Microcephaly, mental retardation	Low IgG, IgM, IgA	N/A on chemotherapy	NBN homozygous c.800dupGp.(Thr268AsnfsTer5)	Sib with same mutation and acute lymphocytic leukemia	N/A	Alive
P18	M	3	5	Acute lymphocytic leukemia	N/A	Recurrent pneumonia, recurrent sinus, prolonged need for antibiotic, failure to thrive (before chemotherapy)	Microcephaly, mental retardation, clubbing	Low IgG, IgM, IgA	N/A on chemotherapy	NBN homozygous c.800dupGp.(Thr268AsnfsTer5)	Sib with same mutation and acute lymphocytic leukemia	IVIG	Alive
P19	M	1.5	3	Acute lymphocytic leukemia	N/A	Recurrent pneumonia, recurrent sinus, prolonged need for antibiotic (after chemotherapy)	Fair-colored hair (positive hair shaft examination for Chediak–Higashi), delayed mental and motor milestones, clubbing	Normal IgG, IgM, IgA	Low CD19	N/A	Negative	N/A	Alive

BMT, bone marrow transplantation; CD, cluster of differentiation; GVHD, graft versus host disease; HLH, hemophagocytic lymphohistiocytosis; HSCT, hematopoietic stem cell transplantation; IVIG, intravenous immunoglobulin; N/A, not available; WASp, Wiskott–Aldrich syndrome protein.

Genetic testing was done on 18/19 patients, and the results were out for 17/19 patients. The diagnosis based on genetic and/or immunological investigation and clinical findings according to the IUIS 2024 classification (22) were as follows: 7/19 (37%) immune-dysregulation, 4/19 (21%) combined immunodeficiency with syndromic features, 3/19 (15.7%) combined immunodeficiency, 3/19 (15.7%) predominantly antibody defect, and 2/19 (10.5%) bone marrow failure defect.

**Figure 2:**
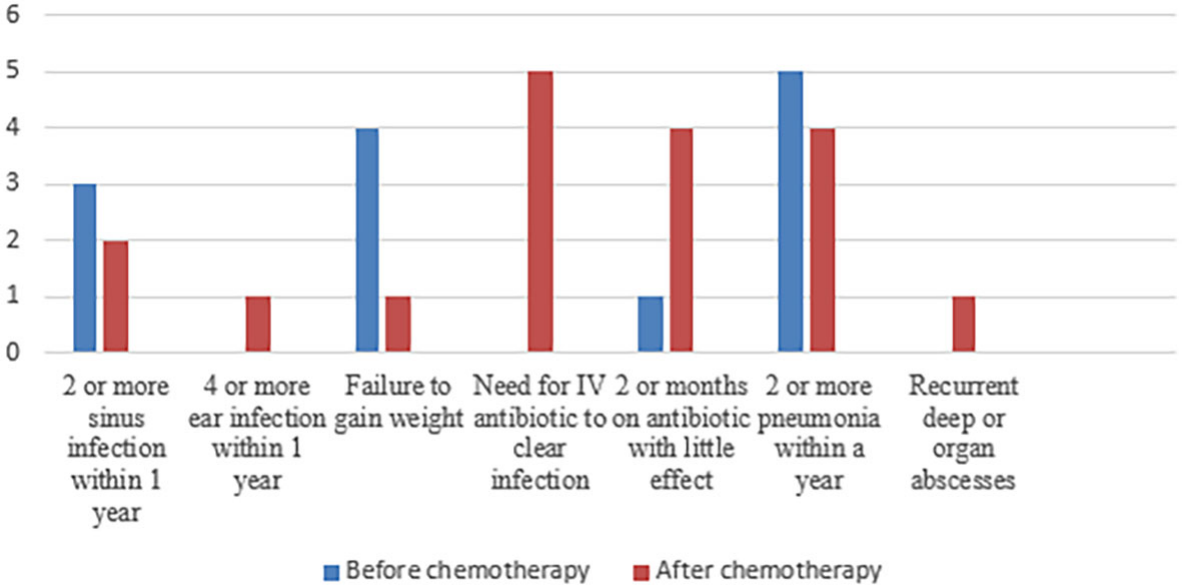
Frequency of the different items of positive 10 warning signs before and after chemotherapy.

As recurrent infection was the main cause of referral, history for the 10 warning signs for primary immunodeficiency was assessed before and after chemotherapy (**Figure 2**). The most frequent warning sign was recurrent pneumonia, followed by recurrent sinus infections and prolonged use of antibiotics. Those infections were either during or after chemotherapy. However, five patients had a significant history of infections before being diagnosed with malignancy, and interestingly, it was not alerting to both parents and their physicians. Those patients had the following diagnosis: P17,18 (combined immunodeficiency and syndromic features (NBN gene mutation) and P4 (immunodysregulation) ITK gene mutation) and P7,14 (predominantly antibody defect (PI3KCD gene mutation) (detailed clinical and laboratory data are shown in **Table 1**).

Regarding the type of malignancy: six (31%) patients had leukemia, either acute myeloid leukemia or acute lymphoblastic leukemia, and 13 (69%) patients had lymphoma, whether Hodgkin’s or non-Hodgkin’s lymphoma (detailed clinical and laboratory data are shown in **Table 2**). All patients received their regular chemotherapy protocol except that P1,4,7 had a dose reduction because of significant chemotoxicity. Two of our patients are still on chemotherapy. One patient died during chemotherapy, 16/19 patients had an initial response to chemotherapy, but unfortunately seven patients relapsed, including P12,13 who had a second relapse after auto-BMT. This makes the overall survival 84% (16/19). As for the adjustment in the treatment plan, nine patients started IVIG, six patients did allo-BMT with a success rate of 83%, three patients were on the BMT list, and four patients refused BMT.

**Table 2:** Comparison between clinical and laboratory data for patients with IEI and leukemia or lymphoma.

Clinical picture/laboratory data	Lymphoma patients	Leukemia patients
Failure to thrive	2/13 (30%)	2/6 (33.3%)
Clubbing	2/13 (15.3%)	3/6 (50%)
EBV positive in tissue/serum	5/13 (33.3%)	0/6
Low Ig	5/13 (33.3%)	3/6 (50%)
Abnormal LSS	10/13 (76%)	2/6 (33.3%)
Family history	6/13 (46%)	5/6 (84%)
Alive	11/13 (85%)	5/6 (84%)
HSCT	4/13 (31%)	0/6

HSCT, hematopoietic stem cell transplantation; Ig, immunoglobulin; LSS, lymphocyte subset.

Lymphoma patients: In our cohort, the patients with lymphoma were nine (69%) men and four (31%) women with a median age of diagnosis of 10 years (2–17 years). Nine patients had Hodgkin’s lymphoma, and five had non-Hodgkin’s lymphoma. Six patients had positive Epstein–Barr virus (EBV) in tissue (three patients in each group). A history of repeated infections was present in five (38.4%) patients. Three patients had a relapse of their original disease, and three patients had significant chemotoxicity. A family history of a sib death due to infection or malignancy or presence of symptoms of immunodysregulation was positive in seven (54%) patients. Interestingly, two sibs with exact ITK mutation (chr5:156667071 G>A; c.852-1G>A) had different clinical and laboratory phenotypes. Seven patients started intravenous immunoglobulin (IVIG) replacement therapy to combat recurrent infections, six patients did HSCT, only one patient died during HSCT from cytomegalovirus (CMV) viremia, and three patients refused HSCT. The overall survival is 11/13 patients.

Leukemia patients: Six patients were diagnosed with leukemia, and they were five (83%) men and one (17%) woman with a median age at diagnosis with malignancy of 5 years (2–9 years). A history of repeated infections was present in five (38.4%) patients. P16 suffered from extensive eczema following treatment with chemotherapy and allo-HSCT from her sib. A family history of a sib who died due to infection or malignancy or presence of malignancy or presence of symptoms of immunodysregulation was positive in five (83%) patients. None of our patients had adjustment in the chemotherapy plan. Only one patient (P15) who did allo-HSCT followed by severe graft-versus-host disease and recurrence of lymphadenopathy is currently controlled on sirolimus. Two patients started IVIG replacement therapy to combat recurrent infections. P14 died from recurrent severe uncontrolled infection during chemotherapy. The overall survival is 5/6 patients.

## 4 Discussion

IEIs have been increasingly recognized in association with hematologic malignancies (23), with a higher incidence of malignancy occurrence early in life (12). Malignancy in IEI patients is considered the second most common cause of death in patients with IEI after infections (24). Knowledge of the fact of increased risk of malignancy in IEI patients highlights the importance of a synergistic effort by immunologists and oncologists in tracking down the potential development of cancer in known IEI patients as well as the possibility of an underlying IEI in patients with newly diagnosed cancers (19).

The possibility of an underlying IEI in patients with newly diagnosed malignancy can be suggested by a medical history of severe infections or a family history of IEI, the type of cancer, age of the patient, or a high rate of therapy-related toxicity (3, 19). Based on our cohort, recurrent infections during the patients’ follow-up along with a positive family history of malignancy or recurrent infection were the most frequent alerting signs for an oncologist to refer the patients for further diagnosis. Adding to that are some clinical clues at first presentation to the oncologist such as clubbing, failure to thrive, persistent eczema, and facies. Other causes were significant chemotoxicity and relapse of malignancy after auto-BMT.

Being a rare disease with a broad spectrum of clinical phenotype, the exact incidence rates for malignancies occurring are difficult to ascertain and confirm (7). The risk of malignancy in IEI has been individually mentioned in different studies, and it showed a range of 1.42–2.3 relatively increased risk in comparison to the general population (10, 16, 18). Unfortunately, we could not screen each patient coming to the hospital for the possibility of IEI, and accordingly the true incidence could not be assessed.

The malignancies most identified in IEI are those related to the lymphoreticular system such as lymphomas, leukemias, malignant histiocytosis, and thymus tumors corresponding up to 96% of the identified malignancies (18). Non-Hodgkin’s lymphoma (NHL) and Hodgkin’s disease (HD) account for 48.6% and 10%, respectively, of the malignancies seen in patients with IEI (25). The presence of a persistent lymphoproliferation in IEI patients makes the diagnosis of lymphoid malignancies very challenging. In addition to the challenge in poor response to treatment protocols, there is an increased risk of toxicity and the contraindication of radiotherapy in some of them such as in DNA repair defect (26). It is also worth mentioning that both NHL and HL are diagnosed at younger ages in patients with IEI, and NHL is more common in men with IEI (5, 6, 27, 28). This went in alignment with our study, where all patients with NHL were male, and the median age of diagnosis was 6 years (1.5–9 years).

Today’s standard of care for patients with clinically diagnosed or suspected IEI involves genetic testing. The different genetic testing methods available include Sanger sequencing of single genes, targeted gene sequencing panels (targeted next-generation sequencing, tNGS), whole exome sequencing (WES), and whole genome sequencing (WGS), which can all be expanded to trio- or whole-family analyses. The choice of the appropriate method depends on the clinical presentation, the suspected type of IEI, and access to resources (29, 30). The goal of genetic testing in IEI is to confirm the clinical diagnosis and accordingly improve the patients’ management. A genetic diagnosis can inform about prognosis, guide in treatment decisions, enable genetic counseling, and provide the opportunity for predictive family testing for relatives at risk. In our cohort, we did WES for 21 patients, and only 18 patients had a confirmatory IEI genetic mutation.

Unlike our study where the most prevalent IEI diagnosis was immunodysregulation, several studies showed a higher prevalence of malignancy in predominantly antibody production deficiencies (31, 32), while others showed more prevalence in the combined deficiencies of T and B cells (ataxia telangiectasia mainly, hyper-IgE syndromes, Wiskott–Aldrich syndrome), activated phosphoinositide 3-kinase delta syndrome (APDS), and autoimmune lymphoproliferative syndrome (ALPS) (19, 33–35).

The treatment of IEI patients with malignancy is often challenging as it requires balancing between increased susceptibility to infection and the additional suppression of the immune system (33). However, to date, the treatment of malignancies in IEI generally is similar to that of the non-IEI patients. Treatment modalities must be tailored on an individual basis (5). In patients with DNA repair defects, radiomimetic agents should not be a first choice, and alkylating substances, such as daunorubicin, etoposide and methotrexate, should be used with dose reduction (14). In B cell lymphomas, regimens that include anti-CD20 monoclonal antibody (rituximab) for short intervals can yield a better advantageous outcome with less toxicity such as those of infections, mucositis, and bone marrow suppression, which are highly observed in IEI patients (36). Importantly, infectious complications should be prevented by regular antimicrobial prophylaxis and monthly intravenous immunoglobulin (IVIG). HSCT seems to be the ultimate curative therapy for many IEI patients (5, 14). In our cohort the addition of monthly IVIG for those with recurrent infections was beneficial in reducing the rate and severity of infection, along with bronchiectasis progression in those who had it. HSCT was discussed with all patients, and only four did a transplantation with a success rate of 75%.

Screening methods for IEI patients presenting with sporadic infections, autoimmunity, autoinflammation, and malignancy are absent, as the 10 warning signs are only useful in screening patients with infections (37). Using this tool as a screening method excluded 20% of patients with IEI, in whom their clinical presentation did not involve infectious diseases. In addition, only three points of the 10 warning signs were found to be beneficial in suspicious cases, and they were a positive family history, need for intravenous antibiotics, and failure to thrive (38). However, in our cohort, the 10 warning signs for primary immunodeficiency were assessed before and after chemotherapy. They were beneficial in considering IEI as an underlying etiology in some patients. The most frequent warning sign was recurrent pneumonia, followed by recurrent sinus infections and the prolonged use of antibiotics during or after ending chemotherapy.

Finally, raising awareness among oncologists and hematologists about the possibility of IEI as a cause of malignancy was a successful point in our center that helped in diagnosing patients with IEI and adjusting their management plan. This model extended to other oncology centers in Egypt. However, larger studies are needed to develop solid warning signs for IEI in patients with immunodysregulation. International efforts which are needed to create an international registry of IEI cases with detailed information on the occurrence of cancer is fundamental to optimizing the diagnostic process and to evaluating the outcomes of new therapeutic options, aiming to improve prognosis and reduce comorbidities (18).

### 4.1 Study limitations

We could not screen every patient diagnosed with malignancy for an underlying IEI, and we could not provide confirmatory genetic sequencing for all patients suspected with IEI.

## 5 Conclusion

Our data present an example for non-infectious manifestation of IEI and the warning signs we used in our center to suspect IEI as a cause of malignancy. The recommended workup to be done upon suspicion includes immunoglobulin and lymphocyte subset at the time of diagnosis before chemotherapy or after 6 months of chemotherapy with close follow-up of infections. Peculiar presentation and family history are very important points in the suspicion of IEI. A history of infections following the termination of chemotherapy should be taken seriously. Genetic sequencing is an important confirmatory test for the diagnosis of IEI in case of an absence of solid criteria for diagnosing IEI.
